# Spectroscopic features of ultrahigh-pressure impact glasses of the Kara astrobleme

**DOI:** 10.1038/s41598-018-25037-z

**Published:** 2018-05-02

**Authors:** T. G. Shumilova, V. P. Lutoev, S. I. Isaenko, N. S. Kovalchuk, B. A. Makeev, A. Yu. Lysiuk, A. A. Zubov, K. Ernstson

**Affiliations:** 1 Institute of Geology, Komi Scientific Center of Ural Division of Russian Academy of Sciences, Pervomayskaya st. 54, Syktyvkar, 167982 Russia; 2 Hawaii Institute of Geophysics and Planetology, University of Hawaii at Manoa, 1680 East-West Road, Honolulu, HI 96822 USA; 3 Faculty of Philosophy I, University of Würzburg, Würzburg, Germany

## Abstract

The state of substances under ultrahigh pressures and temperatures (UHPHT) now raises a special interest as a matter existing under extreme conditions and as potential new material. Under laboratory conditions only small amounts of micrometer-sized matter are produced at a pressure up to 100 GPa and at room temperature. Simultaneous combination of ultrahigh pressures and temperatures in a lab still requires serious technological effort. Here we describe the composition and structure of the UHPHT vein-like impact glass discovered by us in 2015 on the territory of the Kara astrobleme (Russia) and compare its properties with impact glass from the Ries crater (Germany). A complex of structural and spectroscopic methods presents unusual high pressure marks of structural elements in 8-fold co-ordination that had been described earlier neither in synthetic nor natural glasses. The Kara natural UHPHT glasses being about 70 Ma old have well preserved initial structure, presenting some heterogeneity as a result of partial liquation and crystallization differentiation where an amorphous component is proposed to originate from low level polymerization. Homogeneous parts of the UHPHT glasses can be used to deepened fundamental investigation of a substance under extreme PT conditions and to technological studies for novel material creations.

## Introduction

The state of substances under ultrahigh temperatures and pressures now raises a special interest both from the point of view of fundamental questions of the existence of matter under extreme conditions^1–7^, and from the point of view of practical interest with the aim of evaluating them as possible potential new types of materials^8–11^.

Under laboratory conditions, at extremely high pressures, only small amounts of matter are produced, which are limited to particles of micrometer size at a pressure up to 100 GPa, where the synthesis is carried out preferably at room temperature^1–5^. Simultaneous combination of ultrahigh temperatures and pressures can theoretically be accompanied by the formation of specific materials^7^, which still requires a serious study. Among the objects of natural origin, impact glasses represent interest^12–16^, and UHPHT melt-type varieties are of particular interest, since their formation is caused by high pressures (35–90 GPa, up to hundreds of GPa) with fast subsynchronous high-temperature impact (up to 3000 °C and higher)^17–19^. Within the considered problem, the advantage of natural impact glasses is also their much larger volume and preservation of their structure over millions of years.

During our studies of diamond-bearing impactites of the 70 Ma old Kara astrobleme (Russia), we for the first time discovered high pressure post-impact liquation glasses with coesite^20^, which may be interesting for deepened research of their phase state and evaluation of physical properties.

In the context of a large interest in high-pressure glasses as novel promising materials and with regard to strongly limited experimentally produced matter we suggest that the recent find of natural vein-like UHPHT glasses at the Kara astrobleme^20^ could be pioneering. The glasses, which were formed under extreme PT conditions as essentially macroscopic bodies, can be perspective as possible UHPHT materials and/or valuable substance for fundamental study of matter under extreme conditions. With a complex of spectroscopic methods, we examined the natural UHPHT impact glasses to understand specifics of their composition, structure and character of UHPHT memory through 70 Ma age and level of post impact alteration. For the latter we compare the Kara UHPHT glasses characteristics with substantially younger diamond-bearing glasses from the about 15 Ma old Ries impact crater (Germany).

### Geological position and sampling regions

Two impact craters, Kara (ca. 60 km) and Ust`-Kara (ca. 25 km, predicted), are located in the north-east of the European part of Russia, and they belong to the North-Eastern part of the Pay-Khoy Ridge structure (SM 1). The Kara astrobleme is completely set on land surface, while Ust`-Kara is generally at seawater depth. At present the Kara astrobleme is observed as depression in landscape and well expressed in the gravity and magnetic fields^21,22^.

**Figure 1:**
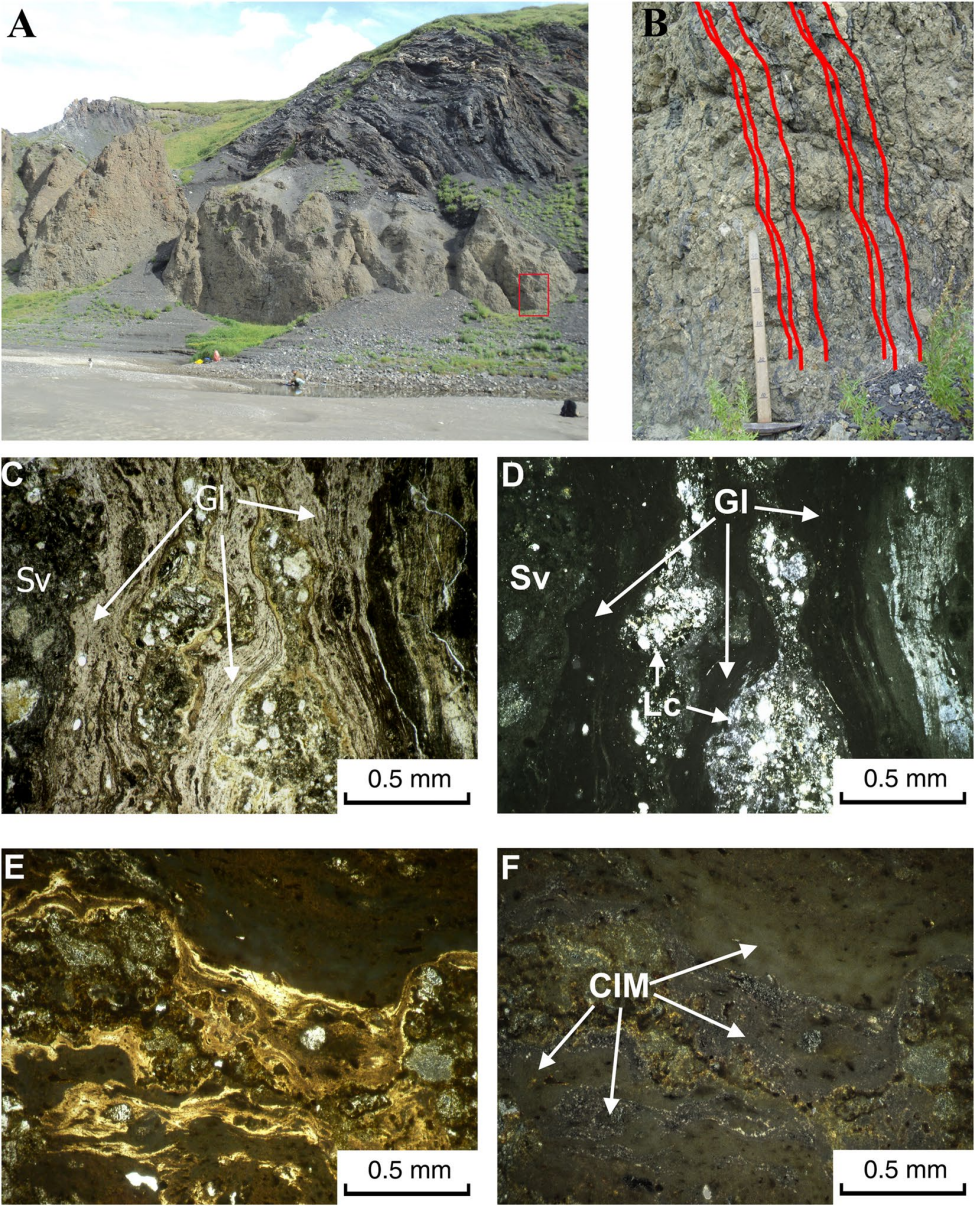
Massive impactite with vein-like ultrahigh pressure melt glasses in contact with black shales of the Kara astrobleme target. (**A**) Outcrop on the right bank of the Kara river, (**B**) magnified part (red square in (**A**)), red lines tracing the vein-like bodies of UHPHT melt glasses. (**C**–**E**) thin sections in transparent light, (**C**,**E**) parallel polarizers, (**D**,**F**) crossed polarizers; (**C**,**D**) UHPHT glass (Gl) in suevite (Sv) transporting lithoclasts (Lc). (**E**,**F**) crystallized impact melt (CIM) clast, CIM clast within suevite. Photos and microimages were made by T.G.Shumilova.

**Figure 2:**
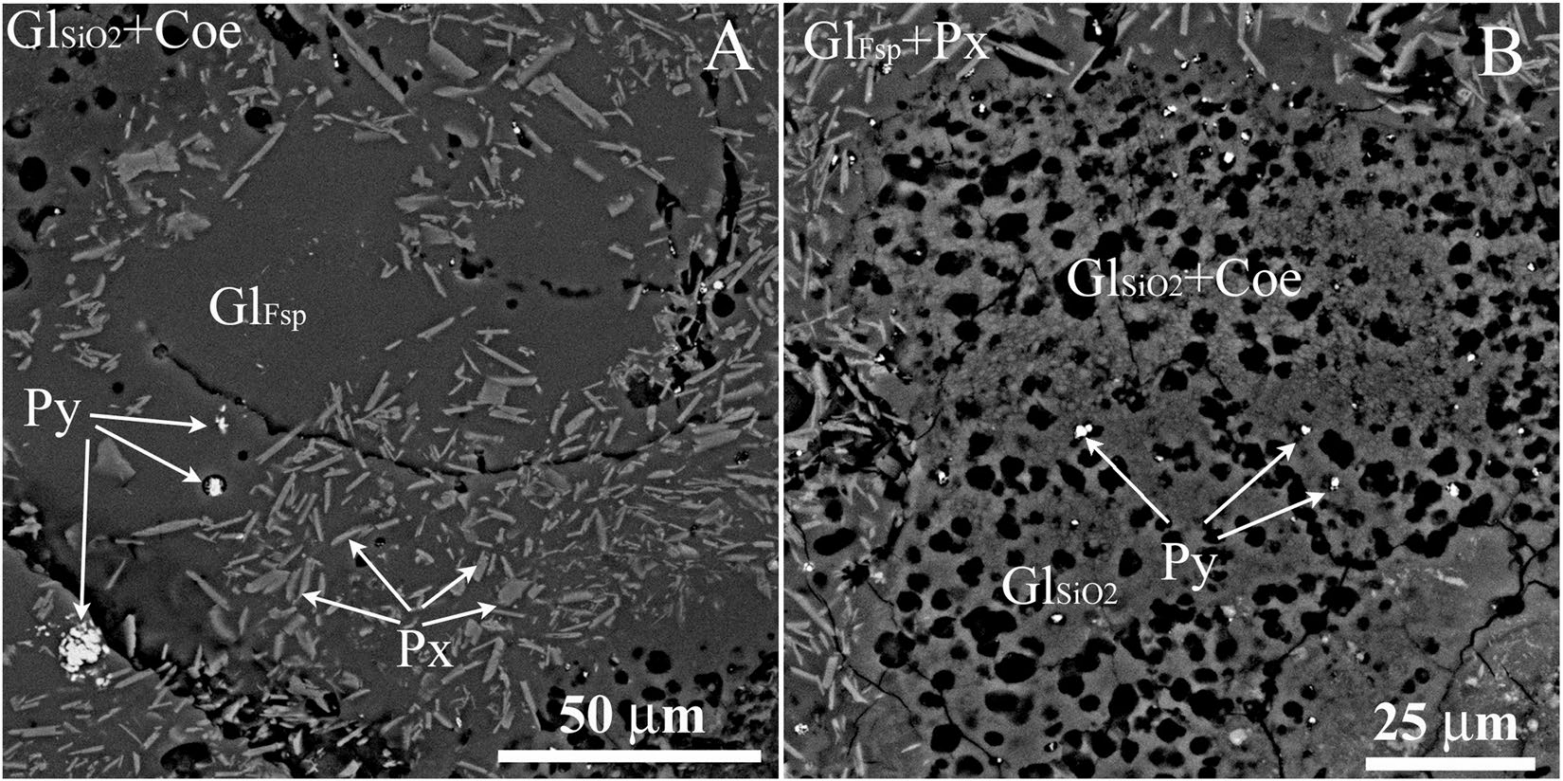
SEM images of different regions in UHPHT impact glass from a vein-like body in the Kara astrobleme, Kr-12–115, Kara river. (**A**) aluminosilicate glass with some pyroxene crystallites, (**B**) coesite-bearing silica glass drop within aluminosilicate glass surrounding matter. Black rounded regions in (**B**) are pores, produced probably at vacuum probe preparing by recovering of liquid (water-rich) inclusions in glass. Gl_Fsp_ and Gl_SiO2_ – feldspar and quartz melting glasses, Coe – coesite, Px – pyroxene, Py – pyrite.

It is proposed that these two astroblemes were formed about 70 Ma past^21–23^ by one bolide, which had been destructed before its contact with the target. The geological structure of the Kara astrobleme is presented by a target consisting of Ordovician-Permian units of black shales, sandstones, limestones and other sedimentary rocks with a total thickness more than 5 km, and by impactites – tagamites (massive melt rocks) and suevites^21,22^. Just this year a complex of vein-like impact melt bodies with coesite has been discovered^20^ (Figs 1 and 2). A unique feature of the Kara astrobleme are abundant aftercoal diamonds^20,24,25^ formed after bitumen coal-like carbonaceous matter, widely spread within the sedimentary rock target.

For the study we collected samples from natural outcrops at banks of the rivers: Kara (suevite, melt rock, vein-like UHPHT glass), Anaroga (clast-poor melt rock) and Sopchau (suevite) at the southern part of the Kara astrobleme close to the outer rim of the impact structure (SM 1).

The about 15 Ma old 26 km-diameter Ries crater in Bavaria (Germany) (also named Nördlinger Ries or Ries Basin) is one of the most studied impact craters on the Earth^26^, which is accepted as a classic middle-size impact structure in a mixed, sedimentary-crystalline target. The geology of the crater and impact glasses have been described elsewhere^26–30^. In this work we used suevites sampled in the Altenbürg and disused Polsingen quarries.

## Results

### Chemical and phase composition of impact glasses

The solidified impact melts of the Kara astrobleme and the Ries crater are in various degree crystallized melts (Fig. 2, SM 2, 3) represented by massive bodies of tagamites; lenses, bombs and ashes in suevites, and also by vein-like formations in massive complexes, recently discovered by us.

**Figure 3:**
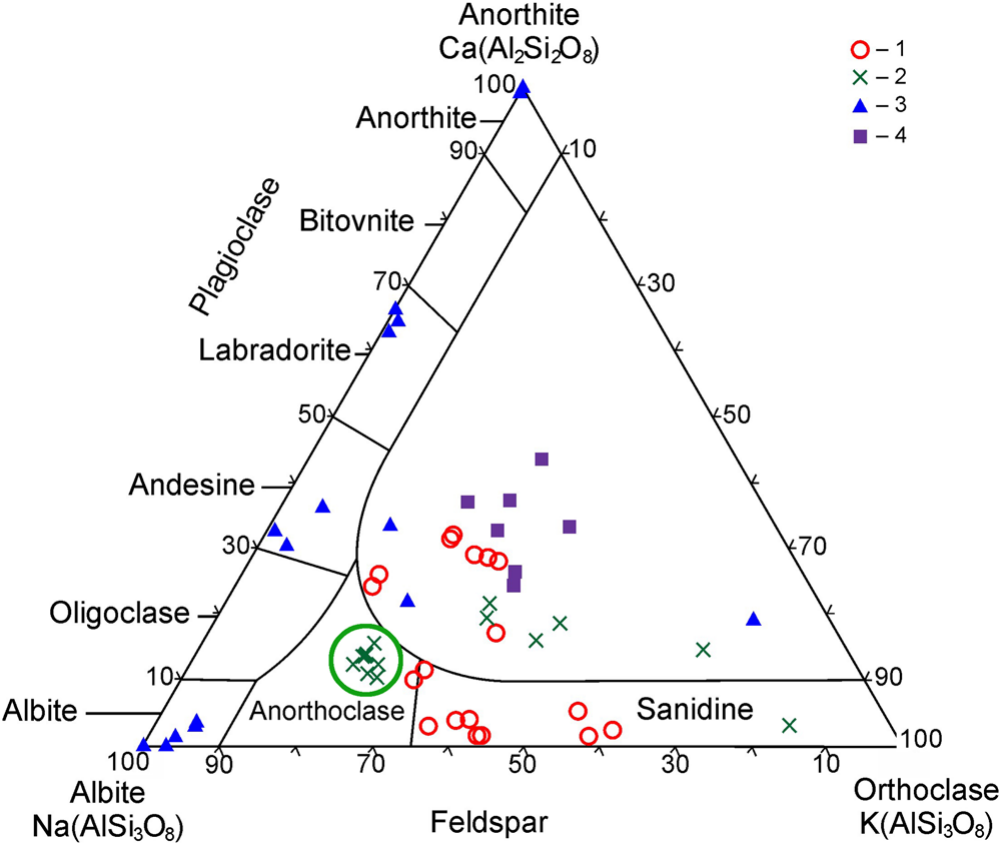
The feldspar ternary diagram of compositions of clastogenic impact glasses from suevites and UHPHT vein-like glasses of the Kara and Ries astroblemes: 1 – Anaroga river; 2 – Kara river; 3 – Sopchau river; 4 – Ries astrobleme, Altenbürg quarry. Points belonging to UHPHT vein-like glasses from the Kara river are encircled in green.

The analysis of the geochemical specificity of impact glasses (IG) and crystallized impact melts (CIM) of the Kara astrobleme and the Ries crater generally showed aluminosilicate compositions (SM 4), which occupy a wide field, covering the regions from basic to ultra-acid composition and a wide range of contents of alkaline components (Fig. 3, SM 5). Such a diversity of impact glass compositions can be explained first of all by the impact melting process having affected a complex lithological interbedding of heterogeneous sedimentary rocks.

**Figure 4:**
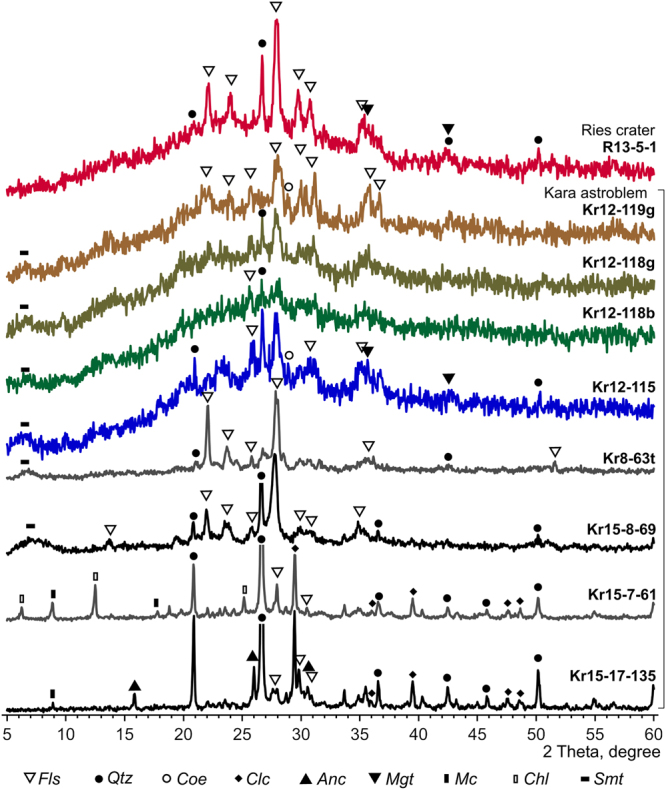
Diffraction patterns of solidified impact melts with different level of crystallinity. Fls – feldspar, Qtz – quarts, Coe – coesite, Clc – calcite, Anc – analcim, Mgt – magnetite, Mc – mica, Chl – chlorite, Smt – smectite.

X-ray diffraction of the impact glasses material from the Kara astrobleme shows an amorphous component of quartz-feldspar glass (Fig. 4). The X-ray patterns show reflexes indicating the presence of feldspar, variously disordered due to the degree of crystallization of the impact melt, as well as of quartz. A weak and strongly broadened reflex corresponds to interplanar spacing 1.4 nm of clay minerals, while a small narrow reflex, corresponding to 0.309 nm be referred to the coesite lattice planes 040.

The diffraction patterns of the glasses from the Ries crater (Fig. 4) are characterized by a similar amorphous halo reflecting the presence of a significant fraction of an amorphous component, and they also have reflexes of feldspar, quartz and weak broadened reflections 0.253 and 0.171 nm, which possibly refer to the reflections from the magnetite lattice planes 311 and 422 (SM 6).

### Local analysis by Raman spectroscopy

**Figure 5:**
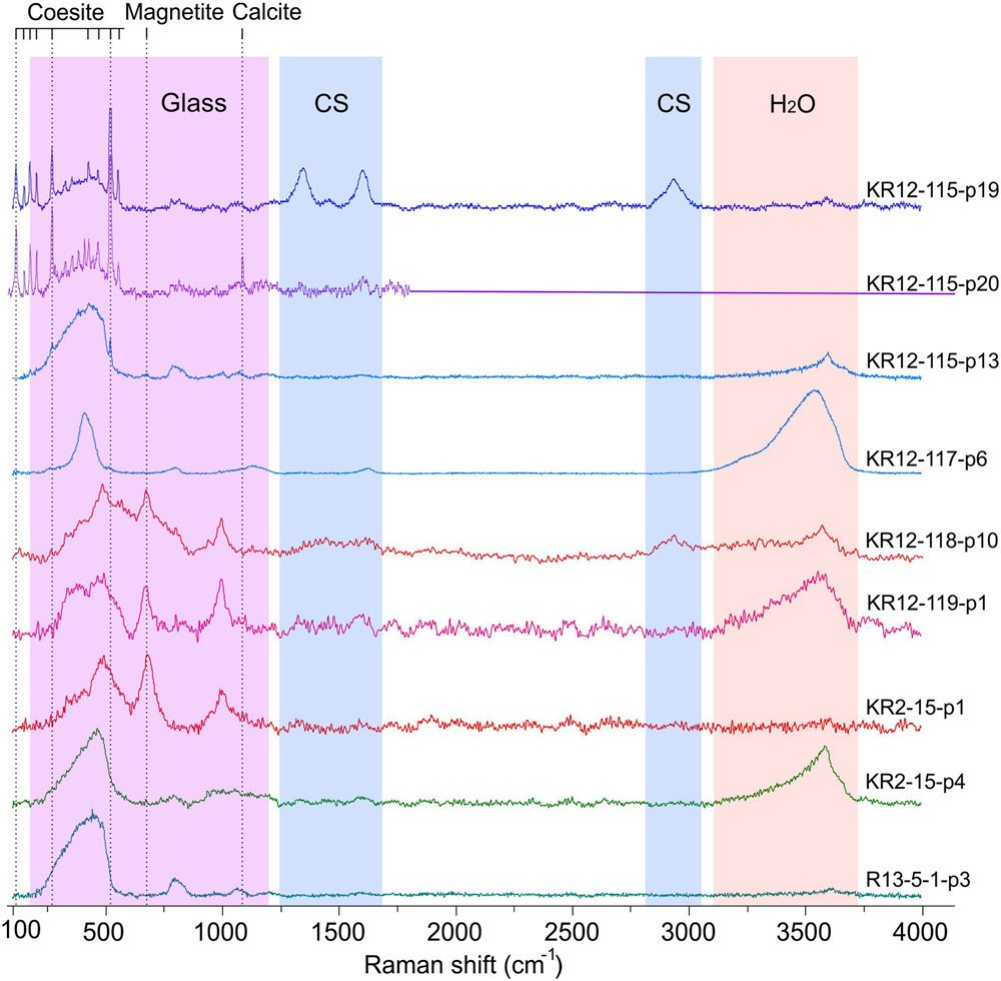
Raman spectra of impact glasses from the Kara astrobleme (KR) and the Ries crater (R). The color marked fields correspond to spectral ranges of glass, carbon and water. Color marked fields – regions of glass (Glass), carbon substance (CS) and H_2_O bands.

Raman spectroscopy of the Kara impact glasses was carried out to identify in detail the components of solidified impact melts from UHPHT vein-like bodies from the Kara astrobleme and the impact glasses of the Altenburg suevites (Ries crater) for comparison. The diagnostics was performed in locally homogeneous areas, where the analysis points were focused on optically visible crystallites and amorphous regions. In both cases the impact glasses proved to be relatively inhomogeneous, characterized by different amounts of microcrystallites of pyroxene and coesite (for the Kara astrobleme) and magnetite (for both astroblemes) (Fig. 5, SM 7, 8). UHPHT impact glasses of the Kara astrobleme are also characterized by the presence of a carbon matter in the graphite-like (glass-like) state or in the state of amorphous carbon - sputtered a-C carbon^31^. At that in a number of cases we determined the co-occurrence of UHPHT silica glass, coesite and carbon matter in a confined volume of only about 1 μm^3^ (Fig. 5, spectrum KR-115-p19, SM 7, 8).

Feldspar glasses (Fig. 5, spectrum KR-117-p6, KR-118a-p10, SM 7, 8) are characterized by different position and structure of the general glass band pointing to variety in polymerization level toward to very small structural elements and their heterogenity. In some cases the maximum band position can be downshifted up to 80 cm^−1^ compare to glass produced at the ambient pressure.

According to the obtained spectra, the UHPHT impact glasses of the Kara astrobleme are characterized by a wide general band, often asymmetric with a broad non-structured or structured shoulder toward a red shift in the spectrum. The general structural band of the impact silica glasses has a full width at half maximum (FWHM) of about 200 cm^−1^ and corresponds to δ(Si-O-Si) bonds of the polymerized structure with a maximum position at 416 cm^−1^, which is substantially shifted from the usual position^32^ at 440 cm^−1^. Unlike glasses, produced at normal pressure, the Raman spectra of UHPHT silica impact glasses are also characterized by the absence of D_1_ and D_2_ bands (490 and 603 cm^−1^ respectively) that refer to the three- and four-membered rings of SiO_4_ tetrahedra in silica glass^32^. These features probably characterize the substantially smaller sizes of the structured regions as a result of the lower degree of polymerization caused by a rapid cooling of UHPHT impact melt.

The feldspar glasses are characterized by differently positioned general Raman bands with similar FWHM to low pressure glasses (about 250–300 cm^−1^). Sometimes the UHPHT feldspar glasses have a complicated general band consisting of several sub-bands with a single FWHM about 100 cm^−1^ in total getting up to 400–500 cm^−1^ (Fig. 5, SM 5, spectrum KR12-118-p10).

The presence of a glass-like carbon matter within the UPH silica glasses is determined by the presence of the bands D = 1347 cm^−1^ and G = 1606 cm^−1^, as well as by a broad band of the second order - 2937 cm^−1^. Sputtered a-C carbon (Fig. 5, spectrum KR12–118-p10, SM 5) is diagnosed according to^31^ by broad bands of 1424, 1608, 2912 cm^−1^, corresponding respectively to D, G and second order bands.

The presence of a large amount of water with a different structural state in glass is noteworthy for UHPHT impact glasses from the Kara astrobleme. The water is predominant in the molecular (absorbed) form, but can partly be chemically bonded to structural radicals in the glass (Fig. 5, spectrum Kr-2-15-p4, SM 7, 8). The Raman spectroscopy allowed us to estimate to some extent the relative water content in UHPHT impact glasses according to the ratio of the integrated intensities of the structural band of the glass-forming component and water. This ratio in the studied material ranges from 0 to 4.5, which indicates a significant inhomogeneity of the fluid in the solidified impact melts. According to experimental data^33^, the water content in UHPHT glasses can make several percent, which conforms with our previous studies using gas chromatography and thermogravimetric analysis^20^.

### FTIR spectroscopy characteristics

**Figure 6:**
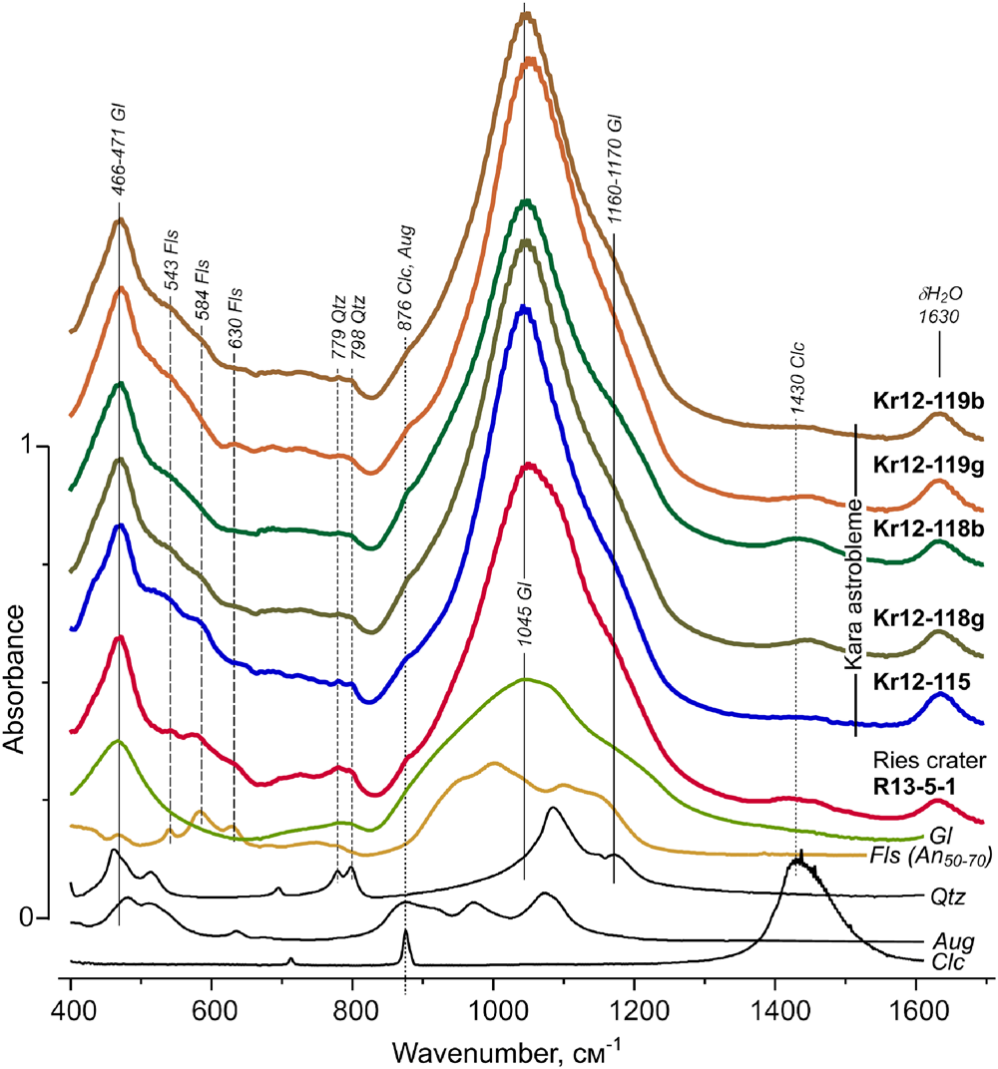
FTIR absorption spectra of impact glasses and standard samples: Fls – plagioclase (labrador An_50–70_), Gl – aluminosilicate glass, Qtz – quartz, Aug – pyroxene (augite), Clc– calcite.

The main features of the FTIR spectra of the samples from the Kara crater and the Ries relate to vibrational modes of framework aluminosilicate melt glasses (Fig. 6). The most intense IR absorption bands are due to the stretching asymmetric (1000–1200 cm^−1^) and bending (∼470 cm^−1^) vibrations of the bridge oxygen of the glass Si-O-Si bonds. In addition a weak broad band at ∼800 cm^−1^ refers to the symmetric stretching vibration of SiO_4_ units of this matrix. Against the background of the last band, very weak traces of the characteristic doublet (779, 798 cm^−1^) of crystalline quartz are noticeable. In the spectra of all glass samples, the high-frequency shoulder of the 470 cm^−1^ band is complicated by weak bands in the region of 540–640 cm^−1^ that relate to the bending vibration O-Si (Al) -O and O-Si-O bands in the plagioclase lattice. The presence of weak bands at 1430 and 880 cm^−1^ in the FTIR spectra indicates an insignificant amount of carbonate and pyroxene in the glass samples.

### Iron state in UHPHT impact glasses by Mössbauer spectroscopy data

**Figure 7:**
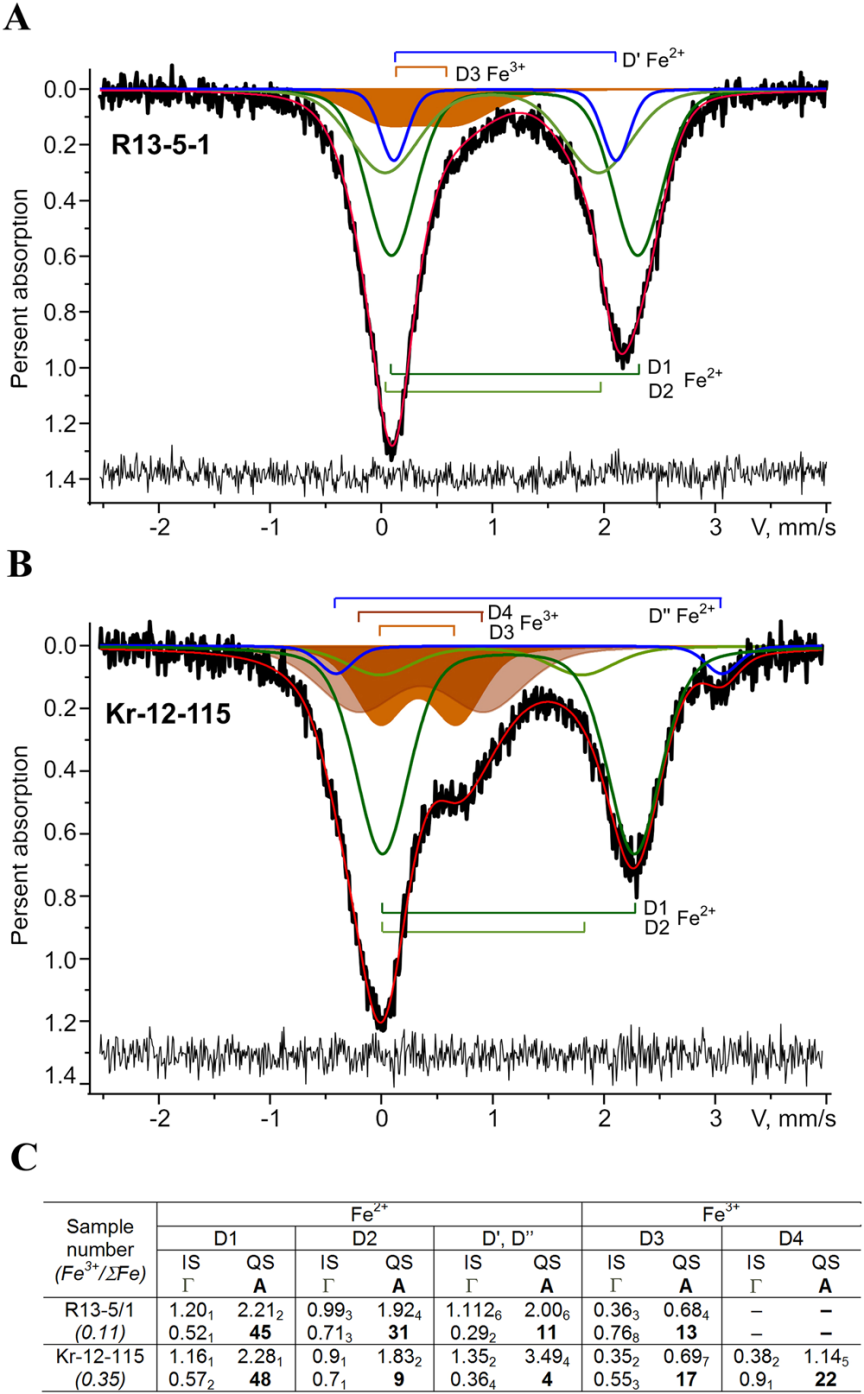
Mössbauer spectra of the impact glasses of the Ries (**A**) and Kara **(B**) craters. Indicated components are the results of the best fits. Residual spectra are shown below. Mössbauer parameters of the best fits components, where IS (mm/s), QS (mm/s), Г (mm/s), A (%) – isomer shift, quadrupole splitting, half-width of doublet peaks, relative area of the doublet. *Fe*^3+^*/*Σ*Fe* = Σ*A*_*Fe*3+_/(Σ*A*_*Fe*2+_. + *k∙*Σ*A*_*Fe*3+_), where k is the ratio of probabilities of Mössbauer transition of octahedral complexes Fe^3+^ and Fe^2+^, k is taken equal to 1.2^32,33^.

The obtained Mössbauer spectra of the impact glasses of both astroblemes contain only paramagnetic doublets (Fig. 7). A sextet structure from magnetic phases is not detected against noise background, although X-ray phase analysis and Raman spectroscopy revealed traces of magnetite in the sample of the glass from the Ries crater (Fig. 4). Analogous spectra were observed in basaltic and meteorite glasses, as well as in tektites^34–38^. The main feature of the obtained spectra is an asymmetric doublet with quadrupole splitting (QS) of about 2 mm/s and isomer shift (IS) of 1 mm/s, typical for Fe^2+^ ions in 4–6 fold oxygen polyhedron sites. The high velocity peak is slightly wider and less intense than the low velocity peak.

As shown by the simulation, the spectra of the Ries crater glass are satisfactorily approximated by a pair of Fe^2+^ doublets (D1, D2) with different values of IS, QS and weak broadened Fe^3+^ doublet (D3). In similar spectra of glass from the Kara crater against the background of the low velocity peak of Fe^2+^ doublets (D1, D2), a broad component with a typically for Fe^3+^ ions small quadrupole splitting is clearly observed. It is a superposition of at least two Fe^3+^ doublets (D3, D4) with different QS values. Besides these broadened doublets related to the glass-like matrix, an additional doublet with narrow peaks (D′), sharpening the main doublets of Fe^2+^, is observed in the spectrum of the glass from the Ries crater. And in the spectrum of the glass from the Kara crater there is a high velocity peak at ∼3 mm/s (D″). The result of decomposition of the spectra in this model and parameters of doublets, obtained in the fitting, are shown in the Fig. 7.

### ESR of impact glasses

**Figure 8:**
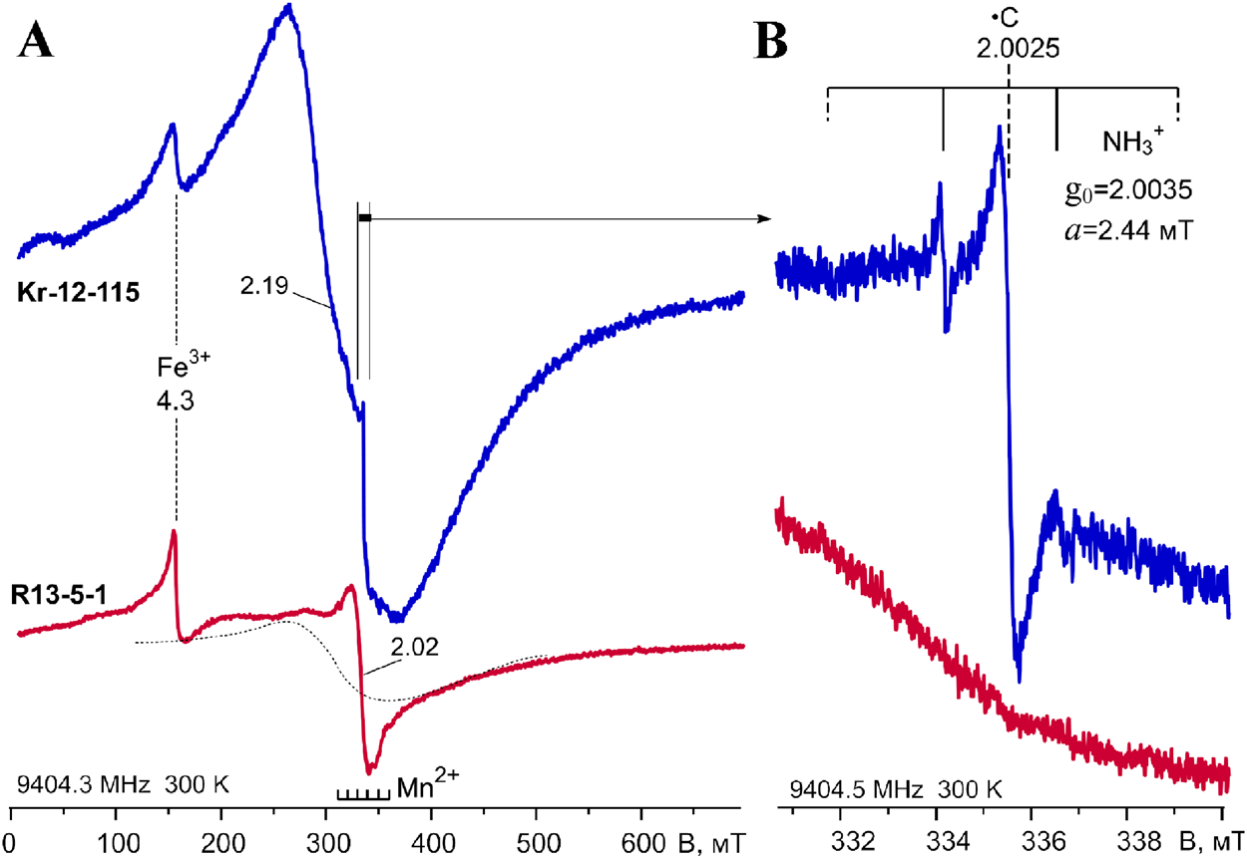
ESR spectra of impact glasses from the Kara astrobleme (upper spectrum) and Ries crater (bottom spectrum): A – the full range spectra; B – magnified part region of g = 2.00. Power of the microwave field is 35 and 7 mW for A and B). The spectra of samples are reduced to the same registration conditions.

The obtained spectra of glasses in the range of the polarizing magnetic field 0–0.7 T are shown in Fig. 8. They contain a wide band with g = 2.2–2.3 (line widths at extremum points ΔB_pp_ = 210 mT), on which narrow lines with g-factors 4.3 and 2.02 (ΔB_pp_ ∼ 8 and 20 mT, respectively) are superimposed, and also a low-intense sextet of Mn^2+^ ions in the lattice of calcite impurities. The ESR spectrum of the Ries crater glass is dominated by narrow lines 4.3 and 2.02 contrasting with a broad band 2.2–2.3 in the Kara crater glass. With a ratio of 10:7 the integral intensity of a signal 4.3 (I∙ΔB_pp_^2^, where I – peak intensity, ΔB_pp_ - width at extremum points) in the spectra of the glasses from the Ries and Kara craters does not differ significantly. In both glasses the wide band is weakly anisotropic, since the shape of its low-field part slightly changes when the tube with the sample rotates in the resonator. Lines 4.3 and 2.02 are isotropic. When the sample is grinded, the anisotropy of the broad band decreases. This signal can be attributed to Fe^3+^ ions in ferrous mineral inclusions (pyroxenes) in glass or clusters of iron ions in the glass framework.

A narrow signal of free radicals with g = 2.00 is also present in the spectra of the Kara samples, the detailed structure of which is shown in the Fig. 8B. The free radical line is formed by a singlet with g = 2.0025–2.0028 (ΔB_pp_ ∼ 0.3–0.5 mT) and a quartet of approximately equidistant lines with different intensities and widths. The singlet line refers to carbon radicals (•C) in the carbon matter. The quartet is characterized by a medium g-factor 2.0035 and a hyperfine splitting 2.44 mT. Structure-analogous signals are often observed in the EPR powder spectra of alkali feldspars and silica with impurities of organic matter. In feldspar spectra, this signal is usually attributed to the ammonia radical^39^ NH_3_^+^, and in the spectra of silica to the methyl radical^40^∙CH_3_. The value of g-factor and hyperfine splitting of the radicals in the Kara glass spectrum allows attributing them to the ∙NH_3_^+^ radical^41^.

## Discussion

The character of the geochemical specificity of the studied impact glasses, determined by overlapping with the most part of volcanic rock fields on the TAS diagram (SM 5), as well as a significant coverage of “forbidden field” for feldspars (Fig. 3) indicates a significant geochemical difference between the impact glasses and CIM glasses of the Kara astrobleme and glasses of volcanic origin^42–45^, characterized by a more homogeneous composition for individual volcanic structures and batch eruptions.

During structural studies we established that the massive bodies of melt impactites, as well as numerous inclusions of bombs, lens-like bodies, and ash material in suevites, are largely represented by substantially crystallized impact melts (Fig. 2A). Therefore, in the context of the study, the embedded real vein-like glass bodies including UHPHT glass with a significant share of coesite are the most interesting features for further detailed study.

The water found by Raman spectroscopy in the impact glass could greatly affect impact melt solidification temperature, lowering it significantly, thereby intensively decreasing the viscosity of the impact melt and, hence, substantially increasing its mobility. Besides, the presence of water can also affect the preservation of impact diamonds. It should also be noted that the water inclusions finding within the UHPHT impact glasses support the hypothesis of the Kara impactites formation at aquagenic conditions^21^. The studied samples of impact glass from the Ries crater generally do not contain water, and the analyzed optically homogeneous glass regions are characterized by almost complete absence of a crystalline component. The position and structure of the general Raman bands are close to ordinary low-pressure aluminosilicate glasses (Fig. 5, spectrum R13-5-1-p3).

Comparing the obtained Raman spectra of impact glasses, it should be noted that unlike the glasses from the Ries crater, the Kara vein-like UHPHT impact glasses are characterized by a significantly greater heterogeneity with respect to the degree of polymerization, which is significantly lower compared to conventional glasses^32^. This heterogeneity of the UHPHT aluminosilicate glass matrix is attributed to drops of pure silica glasses with coesite (Fig. 2B) and carboniferous matter (Fig. 5, spectrum KR12-115-p19).

The FTIR data of bulk specimens do not allow revealing the specific atomic-level structure of the UHPHT impact glasses, including its fine structure. For a more informative analysis local methods of IR spectroscopy with reference to various homogeneous parts of the vein-like type impact glass are required.

From local detailed Raman spectroscopy it is evident that the crystalline quartz and feldspar X-ray reflections and characteristic IR absorption bands rather originate from a certain quantity of relict quartz crystal-clasts and lithoclasts of polymictic sandstones from the target rocks. Raman studies at microlevel do not reveal any newly formed inclusions of quartz and feldspars directly in the UHPHT impact glasses.

The presence of iron in UHPHT impact glasses allows studying some fine features of the glasses structure under the study, since it significantly expands the possibilities of applying sensitive methods, in particular Mössbauer spectroscopy and electron spin resonance. Wide asymmetric peaks from iron sites in the glass and relatively narrow peaks from iron sites of included crystalline phases are characteristic of Mössbauer spectra.

In the Mössbauer spectra of glasses from Kara and Ries craters (Fig. 7), the most intense doublet D1 with nearly equally large widths (0.5–0.6 mm/s) and the contribution to the total area of the spectral contour is characterized by IS, characteristic of 6-fold oxygen polyhedra with Fe^2+^, that is, the octahedral type of sites in the aluminosilicate glass^46–49^. The QS parameter in the glass from the Kara crater is thus significantly higher than in the glass from the Ries crater. Taking into account the dependence of QS on the degree of distortion of octahedral polyhedra^48^, it can be concluded that the Fe^2+^ octahedral sites in the glass from the Kara crater is characterized by relatively low distortion. The second doublet Fe^2+^ (D2) has a smaller QS value (1.8–1.9 mm/s) and a much smaller isomer shift (0.9–1.0 mm/s). A decrease of IS parameter indicates a decrease of the coordination number of oxygen polyhedron Fe^2+^ to 4, that corresponds to the tetrahedral type of the position. For the glass from the Ries crater, the representativeness of the Fe^2+^ tetrahedral sites is three times higher than in the glass from the Kara crater.

The additional low-intense doublet D′ Fe^2+^ in the spectrum of the glass from the Ries crater has a small width of peaks, which is a characteristic of a crystalline phase, and possibly relates to one of the Fe^2+^ octahedral sites of the pyroxene lattice^49^. In the spectrum of the glass from the Kara crater, the doublet D″ with a relatively small peak width has high values of IS and QS (1.35 and 3.5 mm/s). Note that the doublets with the IS and QS large values in our data were observed earlier neither in synthetic nor natural aluminosilicate glasses nor in impact meteorite and tektites glasses^34–38^. Similar values of these parameters were observed for Fe^2+^ ions in 8-fold sites of garnets^48,49^, which lack an independent confirmation among the crystalline inclusions within the Kara glass. Perhaps 8-fold sites of Fe^2+^ in the Kara UHPHT glass directly evolved at the glass network formation under UHPHT conditions.

Isomer shifts of Fe^3+^ doublets (0.35–0.38 mm/s) correspond to octahedral sites of iron within different silica glass and mineral substances^46,49^. A doublet D3 with the same values of IS, QS also presents in the Kara sample spectrum, but it is supplemented by a strongly broadened doublet D4 with a large quadrupole splitting (1.14 mm/s). The areas of the spectral contours of D3, D4 doublets are approximately equal (Fig. 7C). In the case of Fe^3+^ ions, the rising of the QS value indicates increased distortion of octahedral sites for the D4 doublet. For Ries crater glass spectra only one low-intense doublet D3 with a large peak width and QS ∼ 0.7 mm/s is determined. It is known that the Fe^3+^ doublets can be explained by iron both in aluminosilicate glass framework and mineral inclusions, for example, pyroxene. In contrast with the glass from the Ries crater, pyroxene a Fe^2+^ doublet in octahedral sites was not detected in the spectrum of the glass from the Kara crater. For the latter the Mössbauer data show the degree of iron oxidation (Fe^3+^/ΣFe in Fig. 7C) 3 times higher than in the Ries impact glass. It is possible that Fe-ions in pyroxene of the Kara crater glass were oxidized to Fe^3+^ state.

The ESR method detects only the signals from Fe^3+^ ions in iron-containing glass matrix at room temperature. The lines of bivalent iron, due to a very strong spin-phonon interaction, were resolved in EPR spectra only for the specimens cooled to liquid helium temperature. The isotropic lines with g = 4.3 and 2.02 are the characteristics of ESR spectra of various glasses with an impurity of isolated Fe^3+^ ions, including aluminosilicate ones^50–53^. It is considered that these signals are associated with two types of Fe^3+^ ions isolated in the glass framework: (1) g = 4.3 − ions in purely rhombic strong crystal field, E/D = 1/3, D >> hν (D, E − parameters of axial and rhombic component of crystalline fields, hν ∼ 10 GHz); (2) g = 2.0 - ions in a strong axial field E/D = 0). Fe^3+^ ions in the rhombic field relate to strongly rhombic distorted tetrahedral positions and iron ions in the axial field (g = 2.0) − to axially distorted octahedral positions. In the Mössbauer spectra (Fig. 7) the doublet of the tetrahedral positions of ferric iron (IS = 0.2–0.32 mm/s) may have been masked by the intense components of the other iron positions.

The unusual feature of the UHPHT glass from the Kara crater is the tiny presence of carbon and ammonia radicals (NH_3_^+^) described in the ESR data (Fig. 8B). In mineral matrixes, such as K-feldspars, the ammonia radicals are formed usually from NH_4_^+^ cations under ionizing radiation being stable below 200 °C^39–41^. Precursors (NH_4_^+^) of the analyzed ammonia radicals in the Kara crater glasses perhaps were formed by the interaction of nitrogen oxides with organic matter during heating of the target rocks. Following the high temperature impact conditions the carbon radicals were formed during thermal carbonization of the organic matter. As for the paramagnetic NH_3_^+^ radicals, they could have originated under natural radiation after cooling of the vein-like glasses.

## Conclusions

Our studies show that the UHPHT glasses generally consist of an amorphous matter of feldspar composition. Compared to the clastic glasses in suevites, the compositions of the vein-like glasses are localized within a small region in the chemical diagrams having quite stable anorthoclase content and contain small drops of coesite-containing silica glass. A complex of structural and spectroscopic methods presents unusual high pressure marks of structural elements in 8-fold co-ordination that had been described earlier neither in synthetic nor natural glasses.

This comprehensive and complex study for the first time resulted in a rather detailed characterization of the impact glasses of the Kara astrobleme, including a low degree of polymerization of silicate framework on the basis of Raman spectroscopy data. By complex spectroscopic and microscopic data we also determined a significant structure and composition heterogeneity of the UHPHT glasses probably connected with fast partial liquation and crystallization differentiation. In spite of the long post-impact period of about 70 Ma, this type of glasses preserve mostly the initial structure without essential altering over time and under hydrothermal processes. Thus, the Kara UHPHT glasses can be used to deeper fundamental studies by local and high resolution methods for understanding of a substance state under extreme PT conditions.

## Methods

For the study we selected monomineral fractions of impact glasses by a binocular from impactites of Kara astrobleme and Ries crater, sampled during fieldwork in 2013–2017. The analysis of UHPHT glasses of the Kara astrobleme was performed in comparison with glasses from the Ries crater. The analytical works were carried out by the equipment of Center for Collective Usage “Geonauka” of Institute of Geology Komi SC UB RAS.

The preliminary optical observations were performed using POLAM R-312 polarization microscope (LOMO, Russia) with and without an analyzer.

The elemental composition of the glasses was analyzed by microprobe analysis combined with scanning electron microscopy^20,45,54,55^, which is common today and most informative to study natural inhomogeneous systems, including impactites, where the detrital component introduces significant distortions during the study by chemical methods. We used a scanning electron microscope TESCAN VEGA3 (Czech Republic) with Oxford instruments X-Max energy dispersive device, analyst S.S. Shevchuk.

For a high-quality assessment of the phase mineral composition of the impact glasses in volume we used X-ray phase analysis (XRD) with a Shimadzu XRD-6000 diffractometer. The conditions of the survey – CuK_α_, 30 mА, 30 kV, Ni-filter, scanning step 2θ 0.05°, 1 deg/min. We used pounded specimens placed on a flat aluminum substrate. The local analysis of the structure of the impact glasses and the phase diagnostics of its inclusions were performed using a high-resolution Raman spectrometer LabRam HR 800 (Horiba Jobin Yvon). The conditions for recording the spectra were as follows: monochromator grating - 600 g/mm, confocal hole - 300 μm, slit - 100 μm, exposure time 10 sec, number of signal accumulation cycles - 3, power of an excitation Ar + laser (488 nm) 1.2 mW. The size of analyzed regions of the samples was 2.5 μm^2^. The spectra were recorded at room temperature.

To analyze fine features of the structure of impact glasses we used infrared spectroscopy. Infrared spectra were obtained by a FTIR spectrometer Lumex FT-02 in the range 400–4000 cm^−1^ at 256 scans and with an instrumental resolution 2 cm^−1^. The specimens were prepared as pressed pellets of 800 mg KBr and 1.4–1.7 mg of a powder specimen.

Electron spin resonance (ESR) spectra were obtained by a radiospectrometer SE/X-2547 (“RadioPAN”, Poland) in X-frequency range with 100 kHz HF modulation at room temperature of the samples. We used RX102 rectangular resonator with TE_102_ mode. g-Factors were calibrated according to the standard Li^0^:LiF (g_0_ = 2.00229). The samples weighed from 30 to 60 mg and were rubbed in a jasper mortar to the state of “powder”. The recorded spectra were reduced to the same value of the frequency of SHF quantum, amplification and sample weight. In some cases, the averaging of the spectrum for 3–5 scans was performed to reduce noise.

The Mössbauer ^57^Fe spectra were recorded on a spectrometer MS-1104Em in the mode of a thin absorber in the range of −11 to +11 and −4 to +4 mm/s at room temperature of the preparation. Pounded samples (10–20 mg) were used for spectra accumulation. The accumulation time of spectra was from 100 to 200 hours. The isomer shift was determined relatively to α-Fe. When processing the spectra, Univem standard software of the spectrometer was used.

## Electronic supplementary material

Supplementary information

## References

[1] Benmore CJ (2010). Structural and topological changes in silica glass at pressure. Phys. Rev. B.

[2] Bolmatov, D., Brazhkin, V. V., Trachenko, K. Thermodynamic behavior of supercritical matter. *Nature Communications* **4**, Article Number 2331, 10.1038/ncomms3391 (2013). 10.1038/ncomms3331 23949085

[3] Deschamps T, Margueritat J, Martinet C, Mermet A, Champagnon B (2014). Elastic Moduli of Permanently Densified Silica Glasses. Scientific Reports.

[4] Kono Y (2016). Ultrahigh-pressure polyamorphism in GeO2 glass with coordination number 6. PNAS.

[5] Pronin AA (2010). A. Glassy dynamics under superhigh pressure. Phys. Rev. E..

[6] Sato T, Funamori N (2010). High-pressure structural transformation of SiO2 glass up to 100 GPa. Phys. Rev. B.

[7] Guerette M (2015). Structure and Properties of Silica Glass Densified in Cold Compression and Hot Compression. Scientific Reports.

[8] Shultz, M. M. & Mazurin, O. V. Contemporary concept of glass structure and their properties. Leningrad: Science. 200 p. (in Russian) (1988).

[9] Brazhkin VV, Fomin YD, Lyapin AG, Ryzhov VN, Trachenko K (2012). Two Liquid States of Matter: A Dynamical Line on a Phase Diagram. Phys. Rev..

[10] Stebbin sJF, Poe BT (1999). Pentacoordinate silicon in high-pressure crystalline and glassy phases of calcium disilicate (CaSi_2_O_5_). Geophys. Res. Lett..

[11] Xue X, Stebbins JF, Kanzaki M, Trønnes RG (1989). Silicon coordination and speciation changes in a silicate liquid at high pressures. Science..

[12] Impact craters at the Mesozoic and Cenozoic boundary. Ed. Masaitis V. L., Leningrad: Nauka, 191 p. (in Russian) (1990).

[13] Lyutoev, V. P., Lysyuk, A. Yu. Structure and texture of silica of impactites of the Kara astrobleme. *Vestnik IG Komi SC UB RAS*. **9**, 24–32 (in Russian) (2015).

[14] French, B. M. *Traces of Catastrophe*: A Handbook of Shock-Metamorphic Effects in Terrestrial Meteorite Impact Structures. LPI Contribution No. 954. Lunar and Planetary Institute, Houston, Texas. 120 pp. (1998).

[15] Melosh, H. J. Impact cratering – A geological process. Oxford Univ. Press, New York (1989).

[16] Robertson, P. B. & Grieve, R. A. F. Shock attenuation at terrestrial impact structures. In: Roddy, D. J., Pepin, P. O. and Merill, R. B. (eds) Impact and explosion cratering. Pergamon Press, New York, 687–702 (1977).

[17] Langenhorst F, Deutsch A (1994). Shock experiments on pre-heated α-and β- quartz: I. Optical and density data, Earth Planet. Science Letters.

[18] Schmitt RT (2000). Shock experiments with the H6 chondrite Kernouvé: pressure calibration of microscopic shock effects. Meteoritics & Planet. Sci..

[19] Stöffler D (1984). Glasses formed by hypervelocity impact. J. Non-Cryst. Solids..

[20] Shumilova, T. G., Isaenko, S. I., Makeev, B. A. & Zubov, A. A. Liquation features of impact melt under ultrahigh pressure conditions. Abstract volume: 200th Anniversary Meeting of the Russian Mineralogical Society, Saint-Petersburg, St. Petersburg Mining University, 10–13 October 2017 (2017).

[21] Mashchak MS (1991). Morphology and structure of the Kara and Ust'-Kara astroblemes. International Geology Review..

[22] Shishkin, M. A. *et al*. State Geological Map. Scale 1:1000000 (3rd editing). South-Karskaya series. R-41 – Amderma. Report. Saint-Petersburg, VSEGEI, 383 p. (in Russian) (2012).

[23] Trieloff M, Deutsch A, Jessberger EK (1998). The age of the Kara impact structure, Russia. Meteoritics & Planetary Science..

[24] Yezerskiy VA (1986). High pressure polymorphs produced by the shock transformation of coals. International Geology Review..

[25] Shumilova, T. G., Isaenko, S. I., Ulyashev, V. V., Kazakov, V. A. & Makeev, B. A. After-coal diamonds: an enigmatic type of impact diamonds/European Journal of Mineralogy. 10.1127/ejm/2018/0030-2715 (2018).

[26] Pohl, J., Stöffler, D., Gall, H., Ernstson, K. The Ries impact crater. Impact andExplosion Cratering (Ed. by Roddy, D. J. Pepin, R. O. and Merrill, R. B.), Pergamon Press, New York. 343–404 (1977).

[27] Stöffler D (2013). Ries crater and suevite revisited — Observations and modeling Part I: Observations. Meteoritics & Planetary Science..

[28] Stöffler D (1977). Research drilling Nerdlingen 1973: Polymict breccias, crater basement, and cratering model of the Ries impact structure. Geologica Bavarica..

[29] Osinski GR (2003). Impact glasses in fallout suevites from the Ries impact structure, Germany: An analytical SEM study. Meteoritics & Planetary Science..

[30] Osinski GR (2004). Impact melt rocks from the Ries structure, Germany: an origin as impact melt flows?. Earth and Planetary Science Letters..

[31] Ferrari AC, Robertson J (2004). Raman spectroscopy of amorphous, nanostructured, diamond-like carbon, and nanodiamond. Phil. Trans. R. Soc. Lond. A..

[32] Van Tran, T. T. *et al*. Controlled SnO_2_ nanocrystal growth in SiO_2_–SnO_2_ glass-ceramic monoliths. *J*. *Raman Spectros*; 10.1002/jrs.3099 (2011).

[33] Qiang S, Hongsen X, Haifei Z, Jie G, Dongye D (2002). Experimental studies of interaction between water and albite melts. Science in China (Series D)..

[34] Dunlap RA (1997). An investigation of Fe oxidation states and site distributions in a Tibetan tektite. Hyperfine Interactions..

[35] Rossano S (1999). ^57^Fe Mössbauer spectroscopy of tektites. Phys. Chem. Minerals..

[36] Lebedeva, S. M., Eremyashev, V. E. & Bykov, V. N. Investigation of natural basalt glasses by the Mössbauer spectroscopy method. *Electronic scientific information journal “Bulletin of the Department of Earth Sciences of RAS”*. № **1**(21), (in Russian) (2003).

[37] Abdu YA (2005). Mössbauer study of glasses in meteorites: the D’Orbigny angrite and Cachari eucrite. Hyperfine Interactions..

[38] Fern GR, Mather TA, Pyl DM (2005). Investigation of near-source basaltic glasses using ^57^Fe Mössbauer spectroscopy. Hyperfine Interactions..

[39] Matyash IV, Bagmut NN, Litovchenko AS, Proshko VY (1982). Electron paramagnetic resonance study of new paramagnetrc centers in microcline-perthites from pegmatites. . Physics and Chemistry of Minerals..

[40] Ikeya, M. New applications of electron spin resonance: dating, dosimetry and microscopy/copy ed. by Zimmerman, M.R. & Whitehead, N. Singapore; River Edge: World Scientific, 500 p. (1993).

[41] Sasaoka H, Yamanka C, Ikeya M (1996). Is the quartet due to∙CH_3_ and C_2_H_5_ or NH_3_^+^ in alkali feldspars?. Appl. Radiat. Isol..

[42] Albert, P. J. Volcanic glass geochemistry of Italian proximal deposits linked to distal archives in the central Mediterranean region. PhD thesis, University of London (August 2012).

[43] Fujioka, K., Furuta, T. & Arai, F. Petrography and geochemistry of volcanic glass: Leg 57, Deep Sea Drilling Project. In: Scientific Party, Initial Reports of the Deep Sea Drilling Project, 56/57 (eds), Initial Reports of the Deep Sea Drilling Project (U.S. Govt. Printing Office), 56–57, 1049–1066 (1980).

[44] Popov VK, Sakhno VG, Kuzmin YV, Glascock MD, Choi BK (2005). Geochemistry of Volcanic Glasses from the Paektusan Volcano. Doklady Earth Sciences..

[45] Nichols, A. R. L., Beier, C., Brandl, P. A., Buchs, D. M. & Krumm, S. H. Geochemistry of volcanic glasses from the Louisville Seamount Trail (IODP Expedition 330): Implications for eruption environments and mantle melting Geochemistry, Geophysics, Geosystems. 1–21; 10.1002/2013GC005086 (2014).

[46] Dyar MD (1985). A review of Mössbauer data on inorganic glasses: the effects of composition on iron valency and coordination. American Mineralogist..

[47] Menil F (1985). Systematic trends of the 57Fe Mössbauer isomer Shifts in (FeO_n_) and (FeF_n_) polyhedral. Evidance of a new correlation between the isomer shift and the inductive effect of the competing bond *T* − *X* (→Fe) (where *X* is O or F and *T* any element with a formal positive charge). J. Phys. Solids..

[48] Burns RG (1994). Mineral Mössbauer spectroscopy: Correlations between chemical shift and quadrupole spfitting parameters. Hyperfine Interactions..

[49] Vandenberghe, R. E. & De Grave E. Application of Mössbauer Spectroscopy in Earth Sciences. Mössbauer Spectroscopy. Tutorial Book (Ed. by Yutaka Yoshida and Guido Langouche). Springer-Verlag Berlin Heidelberg, 91–186 (2013).

[50] Brodbeck CM (1980). Investigation of g-value correlations associated with the g = 4.3 ESR signal of Fe^3+^ in glass. J. of Non-Crystalline Solids..

[51] Klyava, Y. G. EPR spectroscopy of disordered solid bodies. Riga: Zinatie, 320 p. (in Russian) (1988).

[52] Antoni E (2004). Structural characteriuzation of iron-alumino-silicate glasses. J. of Non-Crystalline Solids..

[53] Dunaeva ES, Ugolkova EA, Efimov NN, Minin VV, Novotortsev VM (2014). ESR spectroscopy of FeIII ions in sodium silicate glass. Russian Chemical Bulletin..

[54] Raschke, U., Schmitt, R. T. & Reimold, W.U. Petrography and geochemistry of impactites and volcanic bedrock in the ICDP drill core D1c from lake El’gyytgyn, NE Russia. *Meteoritics & Planetary Science*. **48**, No. 7, 1251–1286; 10.1111/maps.12087 (2013). 10.1111/maps.12146 PMC4461123 26074719

[55] Rowe MC, Ellios BS, Lindeberg A (2012). Quantifying crystallization and devitrification of rhyolites by means of X-ray diffraction and electron microprobe analysis. American Mineralogist.

