# Supplementary material for: Spectroscopic features of ultrahigh-pressure impact glasses of the Kara astrobleme
Source: Sci Rep. 2018 May 2;8:6923. doi: 10.1038/s41598-018-25037-z (PMC5932052; doi:10.1038/s41598-018-25037-z)
Supplement: Supplementary file 1 — Supplementary information [file 41598_2018_25037_MOESM1_ESM.doc]

**Supplementary information**

**to the manuscript:**

**Title:** Spectroscopic features of ultrahigh-pressure impact glasses

of the Kara astrobleme

**Authors:** Shumilova T.G.^1, 2^, Lutoev V.P.^1^, Isaenko S.I.^1^, Kovalchuk N.S.^1^,

Makeev B.A.^1^, Lysiuk A.Yu.^1^, Zubov A.A.^1^, Ernstson K.^3^

^1^Institute of Geology, Komi Scientific Center of Ural Division of

Russian Academy of Sciences, Pervomayskaya st. 54, Syktyvkar, 167982, Russia;

E-mail: [shumilova@geo.komisc.ru](mailto:shumilova@geo.komisc.ru);

^2^Hawaii Institute of Geophysics and Planetology, University of Hawaii at Manoa,

1680 East-West Road, Honolulu, HI, 96822, USA; E-mail: [tg_shumilova@mail.ru](mailto:tg_shumilova@mail.ru)

^3^Faculty of Philosophy I, University of Würzburg, Germany; E-mail: kernstson@ernstson.de


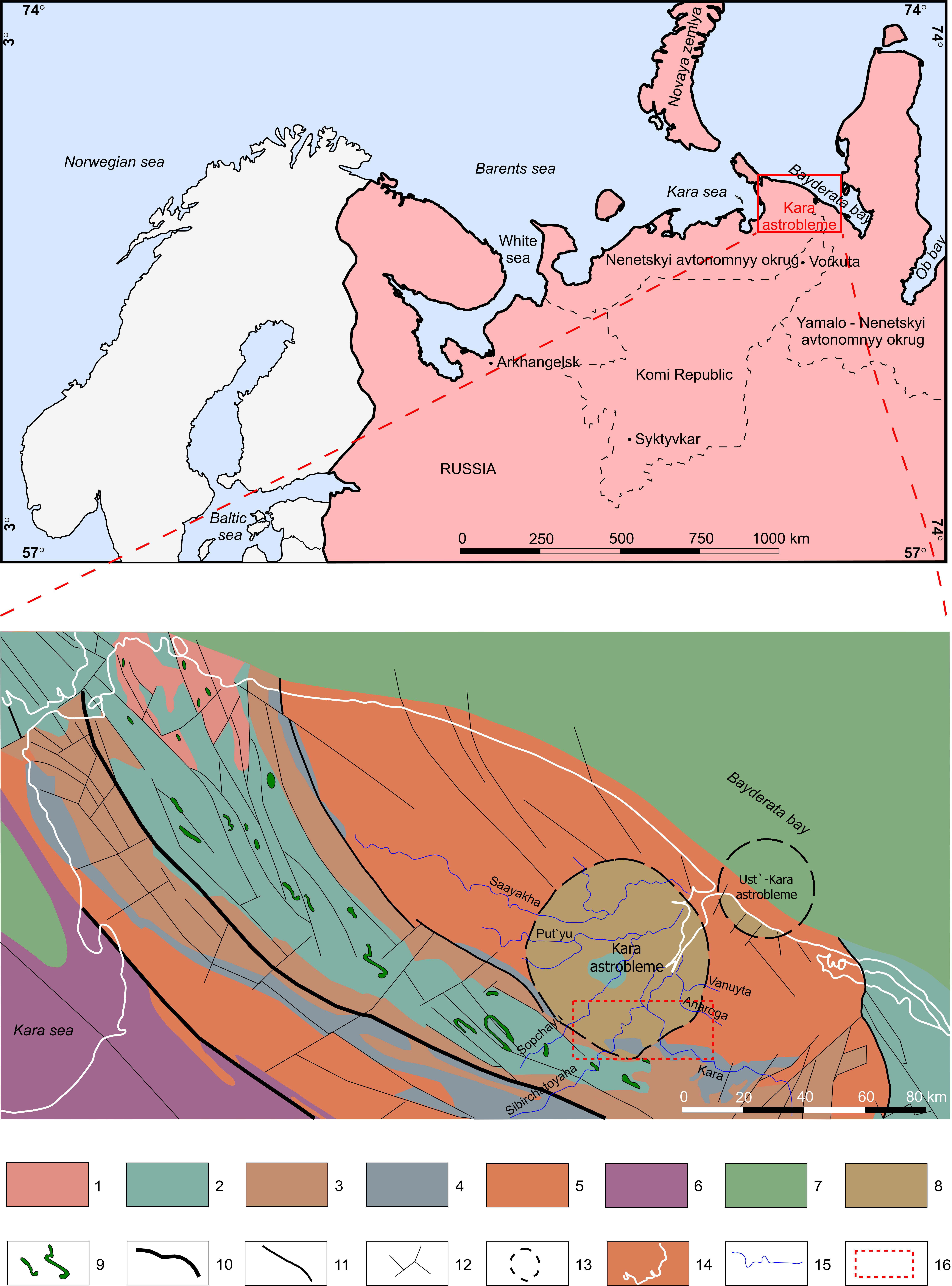


SM 1. Geological scheme of the Kara region territory, simplified by S.I.Isaenko and T.G.Shumilova after the State Geological Map of Russia (GGK-1000) (reference: State Geological Map of Russia (GGK-1000), list R40(42), scale 1:1000000, Saint-Petersburg, VSEGEI, 2000). Inset – geographic setting of the studied area. Sedimentary deposits: 1 – Upper Proterozoic; 2 – Silurian and Ordovician; 3 – Devonian, 4 – Carboniferous, 5 – Permian; 6 – Triassic; 7 – Cretaceous. Impactites – 8. Magmatic intrusions: 9 – Late Devonian tabular body and dikes of dolerite and gabbro-dolerite. Tectonic elements: 10 – deep faults; 11 – thrusts; 12 – small faults; 13 – boundary of astrobleme. Geographic elements: 14 – sea coast, 15 – rivers. 16 – sampling region at the Kara astrobleme.


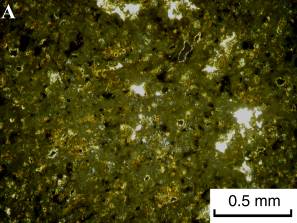

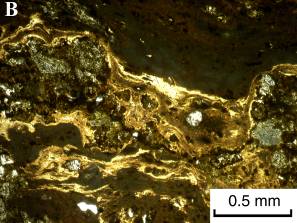


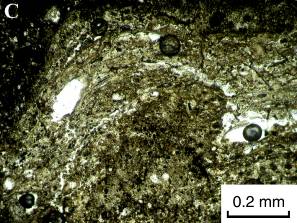

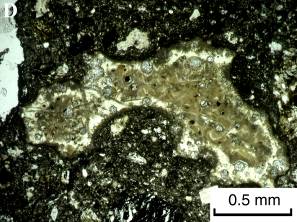


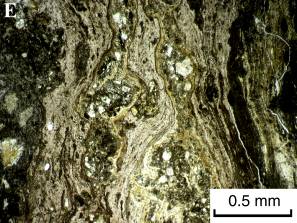

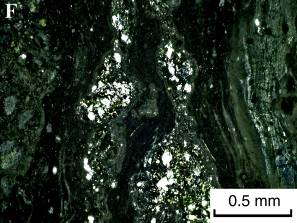


SM 2. Optical images of solidified impact melts of the Kara astrobleme with different level of crystallinity. (A) Tagamite, Kr-8-63, River Anaroga; (B) glass in suevite, Kr-8-71, , River Anaroga; (C) glass in suevite, Kr-7-61, River Kara; (D) glass in suevite, Kr-17-135, River Copcha-U; (E) UHP glass from vein-like body in suevite, Kr-12-115, River Kara (without analyzer), (F) the same with analyzer.


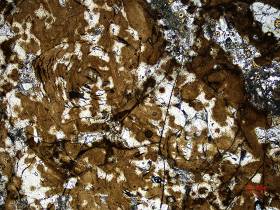

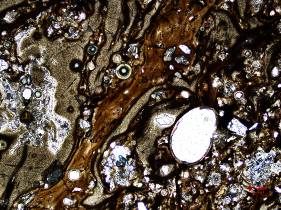


A B

SM 3. Optical images of impact glass of Ries crater, without analyzer. Dark brown regions are enriched with Fe oxides. (A) glass in suevite, R13-5-1, Altenbürg quarry, (B) glass in suevite, R13-8a,

Polsingen quarry.

SM 4. Microprobe data for impact glasses from Kara and Ries astroblemes used for diagrams

| Astrobleme,  region | Analysis  point number | Na_2_O | MgO | Al_2_O_3_ | SiO_2_ | P_2_O_5_ | SO_3_ | K_2_O | CaO | TiO_2_ | MnO | Fe_2_O_3_ | Сумма |  |
| --- | --- | --- | --- | --- | --- | --- | --- | --- | --- | --- | --- | --- | --- | --- |
| Kara  astrobleme,  River  Anaroga | Kr 7-66/2 | 4.07 | 2.45 | 20.65 | 58.71 | 0.23 | 0.00 | 4.30 | 5.14 | 0.94 | 0.00 | 3.51 | 100 |  |
|  | Kr 7-66/3 | 3.63 | 1.91 | 20.67 | 59.61 | 0.30 | 0.00 | 4.64 | 4.71 | 1.20 | 0.00 | 3.33 | 100 |  |
|  | Kr 7-66/4 | 3.95 | 2.09 | 19.94 | 61.49 | 0.00 | 0.00 | 3.41 | 5.15 | 0.87 | 0.00 | 3.10 | 100 |  |
|  | Kr 7-66/5 | 3.77 | 2.01 | 19.79 | 61.71 | 0.18 | 0.00 | 3.29 | 5.07 | 1.04 | 0.00 | 3.15 | 100 |  |
|  | Kr 7-66/6 | 3.88 | 2.12 | 20.53 | 58.72 | 0.23 | 0.00 | 4.58 | 5.03 | 1.22 | 0.00 | 3.70 | 100 |  |
|  | Kr 8-69/1-1 | 6.29 | 0.13 | 20.62 | 64.67 | 0.14 | 0.00 | 5.24 | 2.29 | 0.05 | 0.05 | 0.53 | 100 |  |
|  | Kr 8-69/1-2 | 5.43 | 1.51 | 21.30 | 61.10 | 0.17 | 0.00 | 2.69 | 4.59 | 0.73 | 0.03 | 2.45 | 100 |  |
|  | Kr 8-69/2 | 6.29 | 0.59 | 19.74 | 65.10 | 0.00 | 0.00 | 4.92 | 1.94 | 0.03 | 0.00 | 1.39 | 100 |  |
|  | Kr 8-69/3 | 4.62 | 0.47 | 20.88 | 62.62 | 0.17 | 0.00 | 5.92 | 3.17 | 0.90 | 0.04 | 1.21 | 100 |  |
|  | Kr 8-69/4 | 4.00 | 0.48 | 18.85 | 64.82 | 0.00 | 0.00 | 10.01 | 0.47 | 0.15 | 0.00 | 1.22 | 100 |  |
|  | Kr 8-69/5 | 5.85 | 1.17 | 21.54 | 61.04 | 0.27 | 0.00 | 2.78 | 4.46 | 0.84 | 0.10 | 1.95 | 100 |  |
|  | Kr 8-69/10 | 4.04 | 1.25 | 20.00 | 62.56 | 0.00 | 0.00 | 8.34 | 0.92 | 0.63 | 0.12 | 2.15 | 100 |  |
|  | Kr 8-71а/1 | 5.80 | 0.83 | 21.19 | 63.99 | 0.00 | 0.00 | 6.11 | 0.71 | 0.00 | 0.00 | 1.38 | 100 |  |
|  | Kr 8-71а/2 | 5.42 | 1.24 | 24.49 | 59.78 | 0.00 | 0.00 | 6.12 | 0.71 | 0.00 | 0.00 | 2.24 | 100 |  |
|  | Kr 8-71а/3 | 6.51 | 0.22 | 20.24 | 66.03 | 0.00 | 0.00 | 5.84 | 0.55 | 0.00 | 0.00 | 0.60 | 100 |  |
|  | Kr 8-71а/4 | 5.94 | 0.12 | 19.22 | 66.83 | 0.00 | 0.00 | 7.25 | 0.31 | 0.00 | 0.00 | 0.33 | 100 |  |
|  | Kr 8-71а/5 | 5.71 | 0.82 | 18.98 | 66.00 | 0.00 | 0.00 | 6.80 | 0.28 | 0.00 | 0.00 | 1.41 | 100 |  |
|  | Kr 8-71а/6 | 4.51 | 0.17 | 18.81 | 66.07 | 0.00 | 0.00 | 9.80 | 0.26 | 0.00 | 0.00 | 0.39 | 100 |  |
| Kara  astrobleme,  river Kara | Kr 7-41/1-1 | 3.38 | 1.92 | 19.44 | 60.51 | 0.18 | 0.00 | 6.54 | 3.19 | 1.18 | 0.00 | 3.66 | 100 |  |
|  | Kr 7-41/4 | 3.90 | 2.14 | 19.37 | 60.79 | 0.00 | 0.17 | 6.45 | 2.83 | 0.96 | 0.00 | 3.41 | 100 |  |
|  | Kr 7-41/5 | 4.30 | 1.92 | 20.17 | 59.58 | 0.25 | 0.13 | 5.22 | 3.92 | 1.29 | 0.00 | 3.21 | 100 |  |
|  | Kr 7-41/6 | 4.35 | 1.80 | 20.43 | 60.44 | 0.16 | 0.00 | 5.21 | 3.43 | 1.13 | 0.20 | 2.86 | 100 |  |
|  | Kr 8-61б/1 | 0.84 | 3.08 | 13.56 | 73.58 | 0.14 | 1.80 | 4.46 | 1.17 | 0.33 | 0.00 | 1.05 | 100 |  |
|  | Kr 8-61б/5 | 0.57 | 4.02 | 19.91 | 59.69 | 0.18 | 0.00 | 5.51 | 0.25 | 1.65 | 0.00 | 8.21 | 100 |  |
|  | Kr 12-115/1-1 | 5.85 | 0.28 | 19.07 | 67.18 | 0.00 | 0.00 | 3.10 | 2.25 | 0.42 | 0.00 | 1.86 | 100 |  |
|  | Kr 12-115/1-2 | 4.41 | 3.97 | 17.00 | 62.56 | 0.25 | 0.00 | 2.36 | 1.76 | 0.64 | 0.14 | 6.92 | 100 |  |
|  | Kr 12-115/3 | 4.39 | 5.19 | 17.25 | 61.06 | 0.11 | 0.00 | 2.34 | 1.73 | 0.80 | 0.12 | 7.01 | 100 |  |
|  | Kr 12-115/6 | 4.31 | 4.96 | 18.04 | 60.72 | 0.00 | 0.00 | 2.43 | 1.32 | 0.94 | 0.12 | 7.16 | 100 |  |
|  | Kr 12-115/7 | 4.70 | 4.50 | 18.56 | 60.83 | 0.18 | 0.00 | 2.43 | 1.80 | 0.68 | 0.00 | 6.31 | 100 |  |
|  | Kr 12-115/8-1 | 4.21 | 5.34 | 18.07 | 60.03 | 0.29 | 0.00 | 2.34 | 1.93 | 0.70 | 0.00 | 7.09 | 100 |  |
|  | Kr 12-115/8-2 | 4.61 | 5.01 | 18.95 | 59.16 | 0.18 | 0.00 | 2.26 | 1.56 | 0.73 | 0.00 | 7.54 | 100 |  |
|  | Kr 12-115/8-3 | 4.08 | 5.28 | 18.23 | 60.49 | 0.15 | 0.00 | 2.47 | 1.21 | 0.65 | 0.13 | 7.32 | 100 |  |
|  | Kr 12-115/8-4 | 4.55 | 4.50 | 17.79 | 61.21 | 0.00 | 0.00 | 2.71 | 1.65 | 0.89 | 0.00 | 6.71 | 100 |  |
| Kara  Astrobleme,  River  Sopchau | Kr 17-135/3 | 0.00 | 8.83 | 0.10 | 50.67 | 0.00 | 0.25 | 0.00 | 23.28 | 0.00 | 1.26 | 15.61 | 100 |  |
|  | Kr 17-135/4 | 0.10 | 14.03 | 1.16 | 53.00 | 0.00 | 0.00 | 0.00 | 24.61 | 0.00 | 1.13 | 5.97 | 100 |  |
|  | Kr 17-141/1-4 | 9.74 | 0.00 | 18.21 | 70.88 | 0.00 | 0.00 | 0.56 | 0.24 | 0.00 | 0.17 | 0.20 | 100 |  |
|  | Kr 18-151/1-1 | 7.43 | 2.19 | 17.64 | 62.80 | 0.20 | 0.00 | 0.61 | 6.24 | 1.10 | 0.00 | 1.78 | 100 |  |
|  | Kr 18-151/2 | 6.26 | 1.37 | 17.69 | 63.14 | 0.50 | 0.00 | 4.16 | 4.60 | 0.47 | 0.00 | 1.80 | 100 |  |
|  | Kr 18-151/3-1 | 6.21 | 3.71 | 16.14 | 61.31 | 0.31 | 0.16 | 0.88 | 7.04 | 1.32 | 0.15 | 2.77 | 100 |  |
|  | Kr 18-151/3-2 | 1.04 | 2.30 | 16.61 | 61.06 | 0.00 | 0.00 | 11.26 | 3.64 | 0.86 | 0.16 | 3.06 | 100 |  |
|  | Kr 18-152/1-1 | 4.00 | 6.45 | 11.22 | 55.59 | 0.43 | 0.00 | 0.00 | 14.36 | 0.93 | 0.64 | 6.39 | 100 |  |
|  | Kr 18-152/1-2 | 3.84 | 6.98 | 12.14 | 56.77 | 0.25 | 0.00 | 0.21 | 13.17 | 0.79 | 0.40 | 5.44 | 100 |  |
|  | Kr 18-152/2-1 | 4.30 | 5.89 | 12.39 | 56.45 | 0.19 | 0.00 | 0.16 | 13.56 | 0.65 | 0.33 | 6.08 | 100 |  |
|  | Kr 18-152/2-2 | 7.66 | 1.17 | 18.84 | 62.13 | 0.52 | 0.00 | 0.19 | 6.83 | 1.51 | 0.14 | 0.99 | 100 |  |
|  | Kr 18-152/4 | 4.94 | 1.94 | 16.19 | 65.65 | 0.17 | 0.00 | 2.32 | 5.93 | 0.59 | 0.00 | 2.27 | 100 |  |
|  | Kr 17-141/1-1 | 2.86 | 0.12 | 5.06 | 91.41 | 0.00 | 0.00 | 0.25 | 0.17 | 0.00 | 0.00 | 0.13 | 100 |  |
|  | Kr 17-141/1-2 | 0.11 | 0.00 | 0.17 | 99.59 | 0.00 | 0.00 | 0.00 | 0.00 | 0.13 | 0.00 | 0.00 | 100 |  |
|  | Kr 17-141/1-3 | 1.78 | 0.00 | 3.27 | 94.13 | 0.16 | 0.00 | 0.16 | 0.12 | 0.12 | 0.00 | 0.27 | 100 |  |
|  | Kr 17-141/1-5 | 2.85 | 0.00 | 4.41 | 92.60 | 0.00 | 0.00 | 0.13 | 0.00 | 0.00 | 0.00 | 0.00 | 100 |  |
| Ries  crater,  Polsingen  quarry | R8a/1-1 | 3.19 | 3.25 | 16.05 | 62.44 | 0.00 | 0.00 | 4.56 | 3.57 | 1.18 | 0.10 | 5.65 | 100 |  |
|  | R8a/1-1 | 1.96 | 3.17 | 16.10 | 62.27 | 0.00 | 0.00 | 4.30 | 4.25 | 1.28 | 0.16 | 6.25 | 100 |  |
| Ries  crater  Altenbürg  quarry | R5/1-4 | 1.62 | 7.19 | 15.97 | 57.54 | 0.00 | 0.00 | 2.93 | 4.90 | 1.05 | 0.06 | 8.65 | 100 |  |
|  | R5/1-4 | 2.87 | 3.06 | 16.77 | 63.97 | 0.00 | 0.00 | 4.13 | 3.58 | 0.87 | 0.11 | 4.42 | 100 |  |
|  | R5/1-4 | 2.75 | 8.84 | 15.34 | 54.43 | 0.00 | 0.00 | 2.60 | 4.72 | 0.77 | 0.04 | 10.36 | 100 |  |
|  | R5/1-1 | 2.69 | 2.78 | 15.91 | 64.10 | 0.00 | 0.00 | 3.37 | 4.27 | 0.81 | 0.05 | 6.04 | 100 |  |
|  | R5/1-1 | 2.41 | 3.80 | 16.70 | 60.84 | 0.00 | 0.00 | 3.30 | 4.89 | 0.82 | 0.03 | 7.04 | 100 |  |
|  |  |  |  |  |  |  |  |  |  |  |  |  |  |  |

**
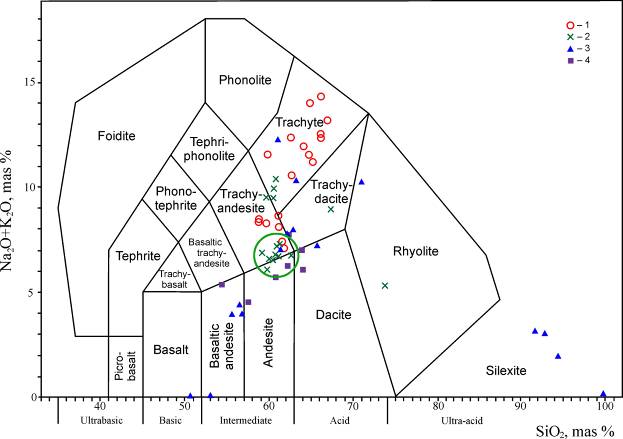
**

SM 5. Impact glasses contents for the Kara astrobleme and Ries crater on alkali and SiO_2_ contents diagram for comparison with volcanic rock composition on TAS diagram: 1 – Anaroga river; 2 – Kara river; 3 – Sopchau river; 4 – Ries crater. Points belonging to UHP vein-like glasses from the Kara river are rounded by a large cycle.

SM 6. Mineral content of solidified impact melts by X-Ray diffraction and FTIR spectroscopy

| Sample number | Deposit | Sample characteristics | Components | |
| --- | --- | --- | --- | --- |
|  |  |  | X-Ray diffraction | FTIR |
| R13-5/1 | Ries crater | impact glass | Fls, Qtz, Mgt? | Gl, Qtz, Fls, Px, Clc |
| Kr12-115  Kr12-118  Kr12-119 | Kara astrobleme | impact glass with coesite from vein-like body | Fls, Qtz, Clm, Coe, Mgt? | Gl, Fls, Qtz, Px, Clc |
| Kr8-63t | -“- | tagamite | Fls, Qtz, Clm | Fls, Qtz(Crs), Px |
| Kr15-8-69 | -“- | impact glass from suevite I | Fls, Qtz, Clm | Fls, Qtz(Crs), Px |
| Kr15-7-61 | -“- | impact glass from suevite II | Qtz, Clc, Fls, Clm | Qtz(Crs), Clc, Px |
| Kr15-17-135 | -“- | impact glass from suevite III | Qtz, Clc, Fls, Anc, Clm | Qtz(Crs), Clc, Px |

Gl – glass, Fls – feldspar, Qtz – quartz, Crs – cristobalite, Goe – coesite, Mgt – magnetite, Px – pyroxen, Clc – calcite, Anc – analcim, Clm – clay minerals (mica, chlorite, and smectite).

SM 7. Raman spectra of impact glasses

**
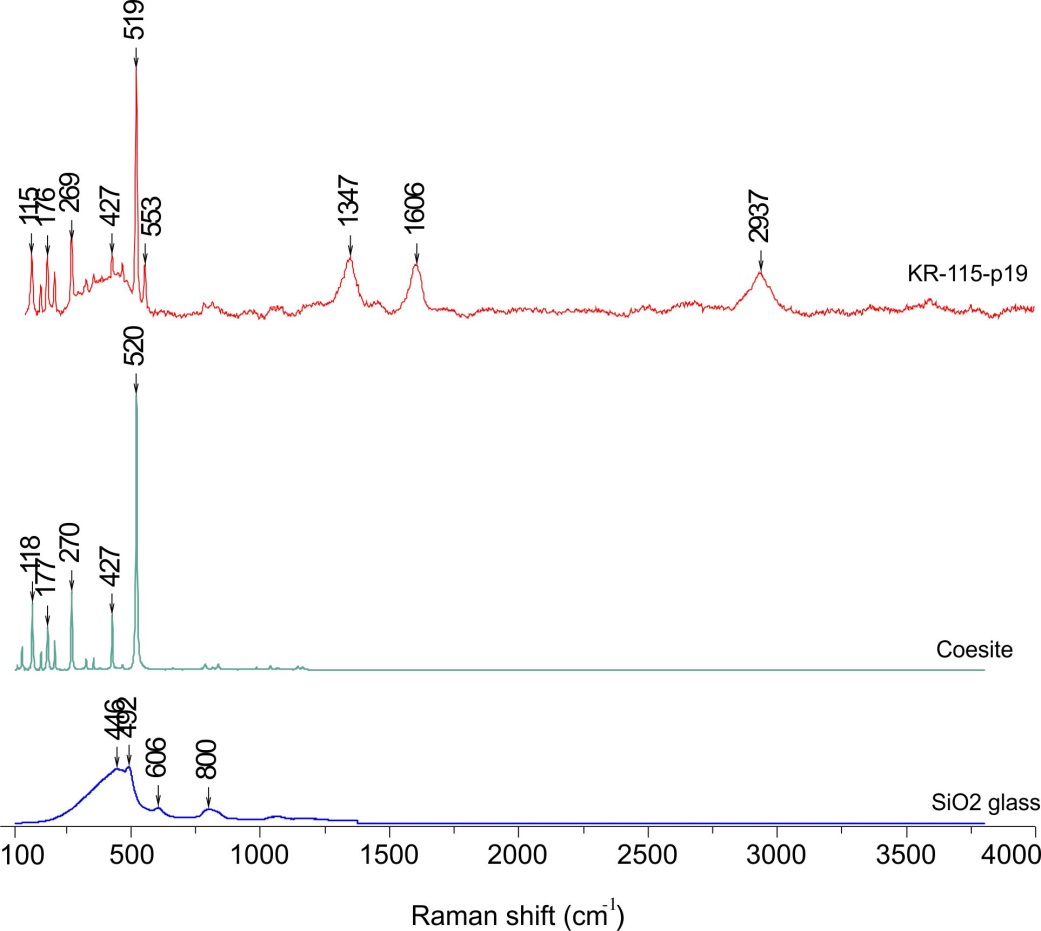
**

Raman spectrum of UHP impact silica glass from vein-like body of the Kara astrobleme with abundant coesite and carbonaceous matter (1347, 1606, 2937 cm^-1^), bottom spectra – standards from RRUFF database for comparison. Decomposed Raman bands description of this and other spectra is presented below in SM 8.

**
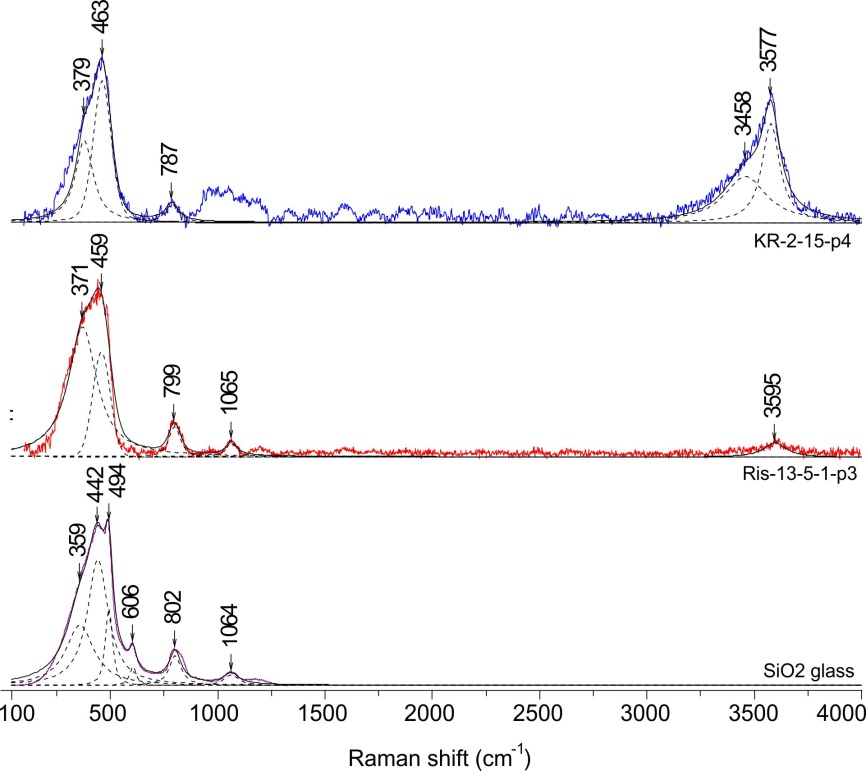
**

Raman spectrum of impact UHP silica glass with high content of absorbed molecular water (3266, 3425, 3542 cm^-1^) from vein-like body of the Kara astrobleme, bottom spectra – standards

from RRUFF database for comparison

**
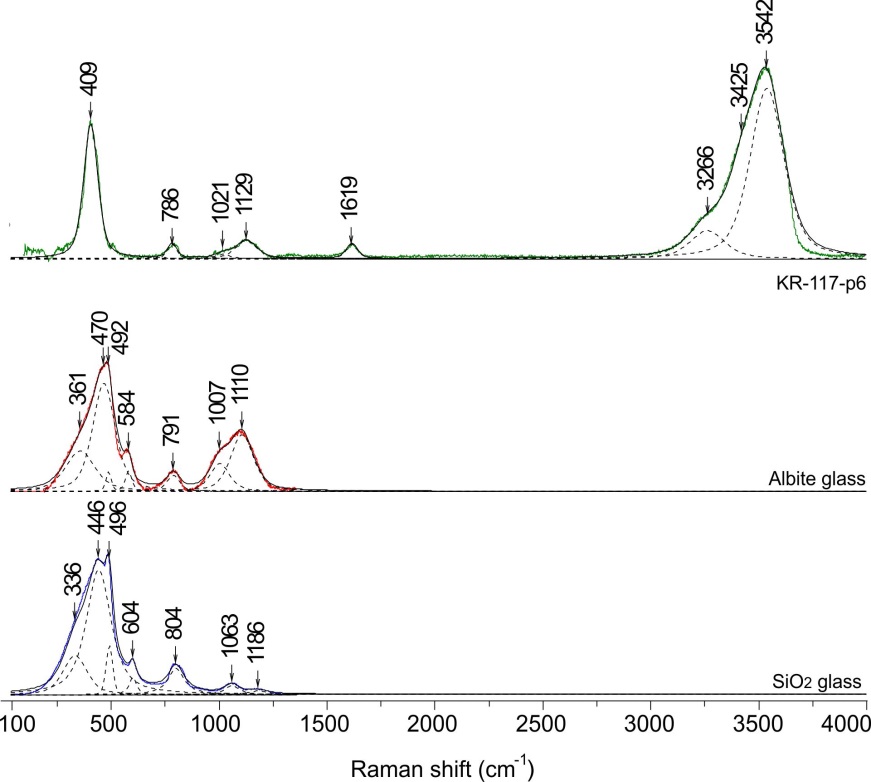
**

Raman spectrum of impact UHP mixture of silica and feldspar glass with high content of absorbed molecular water (3266, 3425, 3542 cm^-1^) from vein-like body of the Kara astrobleme, bottom spectra – standards from RRUFF database for comparison

**
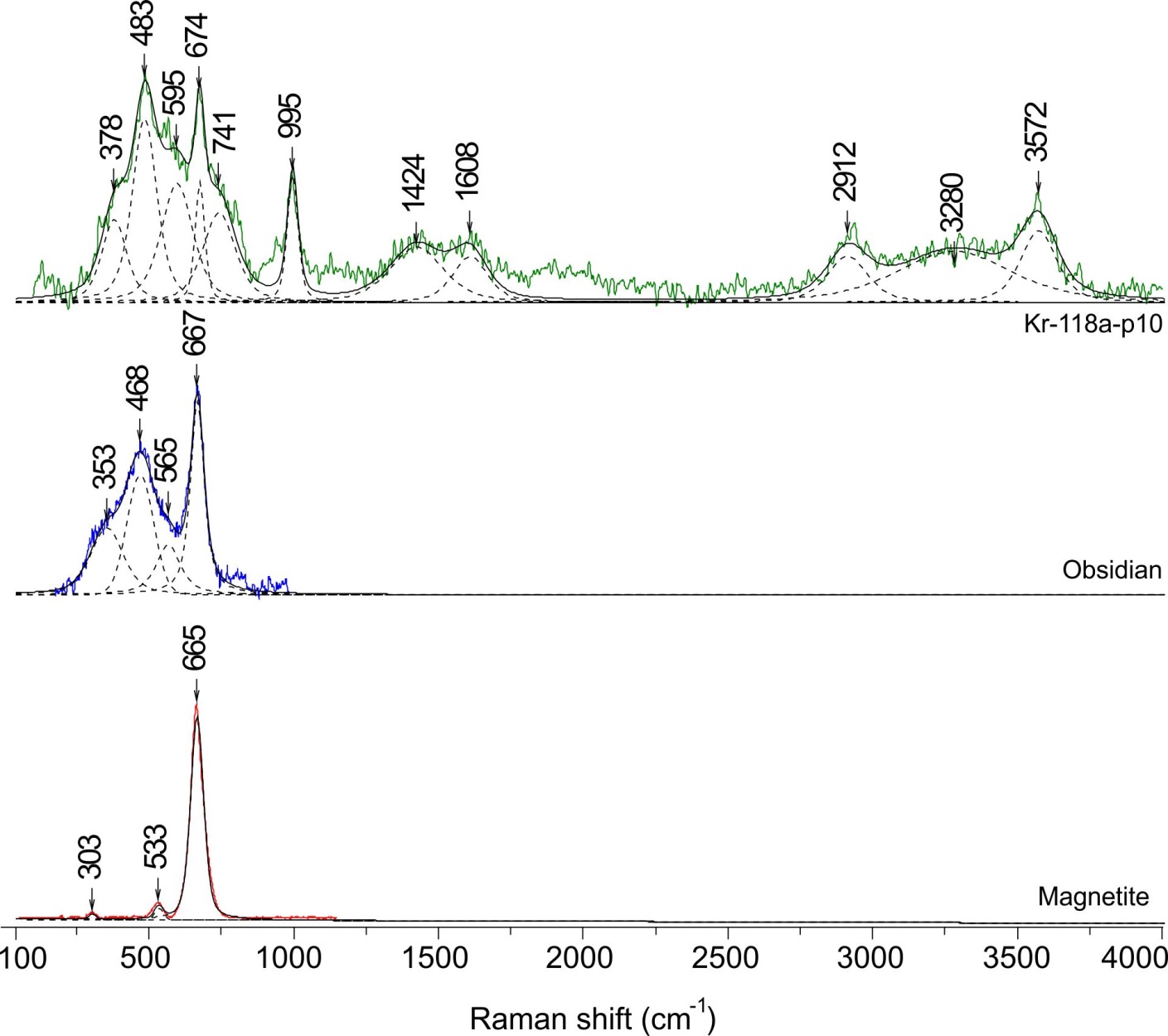
**

Raman spectrum of impact UHP silica glass with magnetite, absorbed water (3572 cm^-1^) and carbonaceous matter, probably diamond-like carbon (1424, 1608, 2912 cm^-1^) from vein-like body of the Kara astrobleme, bottom spectra – standards from RRUFF database for comparison

SM 8 Raman data of the decomposed spectra in SM 7

| Spectrum name | Phase interpretation |  | | Position, cm^-1^ | FWHM, cm^-1^ | Integrated intensity, a.u. | H_2_O/amorph, a.u. |
| --- | --- | --- | --- | --- | --- | --- | --- |
| KR12-115-p19 | coesite |  | | 115 | 8 | 2093 |  |
|  |  |  | | 175 | 6 | 1409 |  |
|  |  |  | | 269 | 8 | 2327 |  |
|  | SiO_2_-glass |  | | 416 | 187 | 26415 | 0 |
|  | coesite |  | | 426 | 6 | 548 |  |
|  |  |  | | 520 | 7 | 977 |  |
|  |  |  | | 553 | 8 | 102 |  |
|  | carbon substance | | 1347 | | 66 | 16843 |  |
|  |  |  | 1602 | | 50 | 8864 |  |
|  |  |  | 2938 | | 91 | 15679 |  |
| KR12-117-p6 | feldspar glass (?) |  | | 409 | 79 | 1868 | 4.5 |
|  | feldspar glass |  | | 786 | 51 | 140 |  |
|  |  |  | | 1021 | 86 | 74 |  |
|  |  |  | | 1129 | 117 | 2 |  |
|  | carbon substance |  | | 1619 | 63 | 172 |  |
|  | water | | 3266 | | 194 | 979 |  |
|  |  |  | 3425 | | 171 | 1792 |  |
|  |  |  | 3542 | | 186 | 5635 |  |
| KR12-118a-p10 | obsidian | | 378 | | 103 | 1223 | 0.5 |
|  |  |  | 483 | | 101 | 2700 |  |
|  |  |  | 595 | | 132 | 2192 |  |
|  | magnetite |  | | 674 | 40 | 704 |  |
|  | obsidian | | 741 | | 133 | 1742 |  |
|  |  |  | 995 | | 44 | 703 |  |
|  | carbon substance | | 1424 | | 222 | 1784 |  |
|  |  |  | 1608 | | 145 | 975 |  |
|  |  |  | 2912 | | 167 | 1123 |  |
|  | water | | 3280 | | 473 | 3175 |  |
|  |  |  | 3572 | | 148 | 1527 |  |
| KR2-15-p4 | SiO_2_-glass |  | | 379 | 100 | 5668 | 1.1 |
|  |  |  | | 463 | 98 | 7746 |  |
|  |  |  | | 787 | 74 | 976 |  |
|  | water |  | | 3458 | 242 | 7508 |  |
|  |  |  | | 3577 | 100 | 6954 |  |
| R13-5-1 | SiO_2_-glass | | 371 | | 161 | 13840 | 0.1 |
|  |  |  | 459 | | 96 | 5012 |  |
|  |  |  | 799 | | 66 | 1226 |  |
|  |  |  | 1065 | | 60 | 609 |  |
|  | water |  | | 3595 | 127 | 1256 |  |
